# Deep disadvantage in mortality on the frontlines of the COVID-19 pandemic

**DOI:** 10.1038/s41598-026-41219-6

**Published:** 2026-04-07

**Authors:** Frank W. Heiland, Jennifer Brite, Deborah Balk

**Affiliations:** 1 Baruch College, New York, USA; 2 CUNY Graduate Center, New York, USA; 3 CUNY Institute for Demographic Research, New York, USA; 4 Hunter College, New York, USA; 5 Marxe School of Public and International Affairs, Baruch College, CUNY Institute for Demographic Research; Faculty, The Graduate Center (Programs in Economics & Demography), City University of New York, 1 Bernard Baruch Way, D-901, New York, NY 10010 USA

**Keywords:** Excess mortality, COVID-19, Unclaimed death, New York city, Disadvantaged populations, Respiratory tract diseases, Risk factors, Viral infection, Health policy, Outcomes research

## Abstract

This study presents new evidence on the temporal and spatial impact of the COVID-19 pandemic on mortality among especially vulnerable New Yorkers. Using burial records from Hart Island—the City’s potter’s field—we study the distribution of unclaimed deaths over time and across boroughs in 2020 compared to pre-pandemic levels. We show that the Hart Island deaths began deviating from their historical pattern in early March 2020 and peaked five weeks later at 22 deaths for every death in the same week in 2019 (20:1 adjusted). COVID-19 excess death rates were more than twice as high in the Bronx compared to other boroughs. Citywide, we estimate that 10% of all COVID-related excess deaths during the initial outbreak (March–August 2020) were unclaimed. These findings suggest the pandemic greatly magnified existing inequalities in the City and, more broadly, illustrate the especially devastating impact of COVID-19 on economically and socially vulnerable populations.

**Supplementary Information:**

The online version contains supplementary material available at 10.1038/s41598-026-41219-6.

## Introduction

While it is well known that New York City (NYC) was the first and hardest hit urban region during the 2020 outbreak of the novel Coronavirus (SARS-CoV-2) in the United States, much about the uneven distribution during this critical early period remains unknown. Between March 11—the first confirmed death from COVID-19, the respiratory disease caused by the SARS-CoV-2 virus—and July 14, 2020, when daily confirmed deaths reached 0 for the first time since mid-March, NYC saw 18,679 confirmed and 4,619 probable COVID-19 deaths. Deaths were heavily concentrated in time and space: Over a period of only seven weeks between the end of March and mid-May mortality soared with the highest daily number of deaths occurred on April 7, with 597 confirmed and 216 probable deaths.^1^ Deaths were concentrated in low-income and immigrant neighborhoods where communities of color were disproportionately affected.^2–9^ By May 13, 2020, towards the end of the first wave of the pandemic in NYC, Black and Hispanics had twice the cumulative age-adjusted rates of confirmed and probable COVID-19 deaths.^10^ Consistent with these racial and ethnic disparities and socioeconomic inequality across the City, the official death rates were also substantially greater in the Bronx and Queens than in the more affluent borough of Manhattan.^1^ Similarly stark disparities in COVID-related outcomes were observed nationwide as the pandemic swept across the US^11–14^

Understanding how COVID-19 has affected disadvantaged populations is an important public health priority. While research on who is at risk of becoming an unclaimed death is emerging^15–18^, it is generally recognized to be a marker of deep economic deprivation and/or social isolation. Communities of color and the poor were disproportionately impacted through disrupted social networks—another consequence of the COVID-19 pandemic and a marker of poor health and premature death^19^—and this may have further compounded who got sick and received treatment or care. Official statistics do not report COVID-related mortality by income (let al.one any marker of isolation, such as family structure), and data by race/ethnicity may suffer from quality issues including high incidence of non-response/missing information.^20^ In the early months of the pandemic, lack of testing, test quality (false negatives), and misclassification of cause-of-death^21–23^ among disadvantaged and isolated groups would also differentially affect the quality of the available information and render official statistics less informative. Knowing that there is large vulnerability within the City, and that official statistics may gloss over such variation, use of alternative data sources to illustrate the magnitude of impact of the outbreak is important and can supplement official statistics.

This paper provides new evidence on the overall impact the first wave of pandemic had on mortality in NYC using burial records from Hart Island—the City’s potter’s field or unclaimed burial grounds. We study the distribution of those interred on Hart Island (herein, Hart Island deaths or unclaimed deaths) by date of death to understand how mortality among some of the most vulnerable New Yorkers changed during the pandemic across the five boroughs. (Boroughs are the equivalent of counties in NYC.) We compare the temporal and spatial dynamics of Hart Island deaths in 2020 to pre-pandemic levels, and place those in comparison to the City’s death rates in the same periods. We examine Hart Island mortality patterns and generate estimates of COVID-related excess mortality, i.e., the difference between the mortality observed and expected in the absence of the pandemic.

Excess mortality is an important metric to better understand the full impact of the pandemic on mortality and population health.^22–28^ Official COVID-related death counts may understate the true number of deaths due to lack of testing and misclassification of cause-of-death.^21–23^ In addition to a direct impact of COVID-19 illness on mortality, excess mortality may also capture changes in other causes of deaths that changed independently or as a result of COVID-19. Individuals with chronic or acute health conditions may be more likely to die during the pandemic because the healthcare system is overwhelmed, treatment is unavailable, inadequate or delayed, and pre-existing conditions make them more vulnerable to COVID-19^24^. Work-from-home may decrease mortality from certain accidents, while unemployment and social isolation may lead to more anxiety and “deaths of despair” (mortality from suicide, accidental drug overdose, and alcohol-related diseases).^23–26,29^ It is considered a feature of the excess-mortality metric that it captures both direct and indirect causes of death.^28,30^.

### Background on unclaimed deaths and why an understanding of them is important

The burial records from Hart Island provide a unique window into the impact of COVID-19 on mortality in NYC. The island has been used as the City’s public cemetery since 1869 (but inaccessible even to family members of the decedents until 2014). It is estimated that more than a million individuals are buried there in mass graves of 150 adults once dug by prison laborers from Rikers Island (as of 2020, prisoners no longer perform burials).^31–33^ Partly due to advocacy by the *Hart Island Project*, a non-profit seeking more transparency of NYC burial procedures, documenting the history of Hart Island and the memories of individuals buried there, and assisting individuals in gaining access to graves and information, the City now provides a searchable online database and opened the island to the public in late 2023 under the new management of NYC Parks and Recreation.

Although some of those interred on Hart Island are unidentified, the majority are able to be identified, and a thorough investigation after a decedent’s death is conducted by the City of New York (generally through the Public Administrator^34^ if no will can be found) to locate next of kin, such as spouses, children, siblings, or parents. Burial on Hart Island may occur if no next of kin can be identified or they cannot afford burial costs, or if they prefer a burial on the island for a variety of reasons, such as estrangement or stigma around cause of death (e.g., COVID-19 or HIV/AIDS)^32,35^. As of 2019, the Human Resources Administration’s (HRA) Office of Burial Services is responsible for facilitating burials for the unclaimed.^36^ The overwhelming majority of New York City unclaimed dead are buried on Hart Island, but sometimes charities and other organizations, or county-local government, step in to provide burial (this is particularly true on Staten Island; see limitations section below).

During the peak of the outbreak in April 2020, Hart Island caught national attention when drone video footage and satellite pictures of mass burials on the island emerged on social media.^37^ At that time, the medical examiner’s office shortened the time until unclaimed bodies in storage were buried on Hart Island to 14 days.^37,38^ Hart Island may not be the final resting place for all those buried there. Sometimes family members request disinterment. Based on our (in-person) inspection of the City’s 2018–2020 paper records only a small number of reinterments had occurred as of 2023. Historically, one source reports an average of 72 disinterments per year from 2007 to 2009.^39^.

Estimating the impact of the COVID-19 pandemic using excess mortality among those who were unclaimed and received a Hart Island burial is novel and important. Unclaimed deaths provide a unique window into mortality among individuals expected to be from particularly economically and socially vulnerable populations. Compared to New Yorkers nearing the end of their life, they may be particularly frail in health, lack timely access to healthcare, and face greater risk of misclassification upon death as they lack social and material resources such as family members who could provide for them economically and who could make decisions on their behalf.^18^ It is also likely that this population disproportionately contains members of the low-skilled “essential worker” workforce (just under 43% of the sample was below age 67 at the time of death), who were particularly affected by greater risk of mortality during the COVID-19 pandemic.^40^ At the same time, they may be omitted from other more standard data collection approaches like sample-survey data collection and analysis.^41,42^ Thus, understanding the toll exacted upon this population, in the first wave of the pandemic when there was much greater uncertainty (little understanding of transmission and etiology and limited treatment options), is particularly important to better understand the disparities in mortality during the COVID-19 pandemic and also to prepare to protect isolated and economically deprived individuals in future pandemics and from other emerging diseases.

## Data and methods

Our analytical strategy is to study COVID-related excess mortality by comparing observed HI deaths during three key periods of the initial outbreak – epi-year 2020, epi-weeks 10–35, and epi-week 15 (peak) – to counterfactual deaths, that is, unclaimed deaths expected in those periods in the absence of the pandemic. We employ three methods to obtain those counterfactuals from pre-pandemic records, ranging from simple comparison and extrapolation (Approaches 1 & 2) to regression-based predictions (Approach 3). Specifically, Approach 1 (A1 in Tables 2 and 3) compares deaths in the respective epidemiological periods in 2020 to 2019; Approach 2 (A2) adjusts the 2019 values for potential pre-pandemic linear trends using the observed differences in deaths between 2019 and 2018; and Approach 3 (A3) predicts counterfactual deaths in 2020 from count data regression models that explicitly control for seasonality, weather, holidays, and general period effects estimated on 2015–2019 data adopting specifications similar to those found in a long-term study of Hart Island mortality (Heiland et al. 2026^43^). The details of this model-based approach including commentary about model fit and overdispersion analysis (to assess whether the data supports use of the more general Negative Binomial model to the standard Poisson count data model) can be found in the Appendix. All three approaches also stratify by borough and sex. These different approaches and comparative time periods produce a robust set of counterfactuals, described further below.

To place COVID-related excess mortality among the unclaimed in the context of excess mortality overall, we replicate the analysis using all (daily) deaths city-wide (Table 4); we do not have borough-specific city-wide daily death data.

We provide 95% Confidence Intervals (CIs) for all results related to the excess deaths calculations. The key patterns and differences that we report in the main text are statistically significant as indicated by those CIs. The underlying Standard Errors and 95% Confidence Intervals are shown in extended results tables reported in the Appendix.

### Study population

This paper focuses on unclaimed deaths in New York City in 2020 based on publicly available burial records from Hart Island, maintained by the City of New York.^31^ Case information includes first and last name, sex, date of death (day), age at death (in completed years), location of death, burial plot, and medical examiner case number (or ME #). Child burials are included in the database, but, during the period considered here, the youngest deceased person was 18 years old. (Burials of infants/fetuses are recorded separately and excluded from the analysis.)

### Data preparation

We cleaned and prepared for analysis more than 8,000 Hart Island burial records from 2015 to 2021. This entailed extensive data quality checks (for completeness and accuracy) using time-intensive comparisons of records retrieved from the City’s online look-up service to the original entries in the Hart Island interment books kept at the offices of the NYC Human Resources Administration. Access to those wide-ledger handwritten logs was made possible through requests to the City of New York under the Freedom of Information Law. We were able to verify and amend records with initially missing or incomplete information in data fields important for the analysis. Specifically, we found several duplicate entries in the publicly available data and infants who were listed under the adult section of the website. In addition, we found several decedents who were missing from the publicly available records and added them. Information on sex was missing for 1,943 individuals, with 33 of them having names listed as “unknown.” For 1,908 of the 1,910 individuals with name information, sex was imputed (male/female) using NamSor^44,45^, an AI tool that uses first name, last name and country of residence to predict gender, and checked via visual inspection of each AI-assigned sex against the available first name. Information on age at death was missing in 42 cases. In total, for our period of analysis, epi-years 2015–2020, there are 6,683 individuals who died and were buried on Hart Island. Analyses by sex are based on as many as 6,648 unclaimed deaths; statistics using age at death are based on as many as 6,651 records. Age values are incremented by 0.5 year to adjust for completed year reporting. (See Brite et al. (2025)^18^ for a more general review of issues in public burial data retrieval and standardization, nationwide.)

Aggregated (daily) city-wide death counts by sex were obtained from the New York City Department of Health and Mental Hygiene Bureau of Vital Statistics. These deaths include all deaths with no distinction between claimed and unclaimed.

This research program has been reviewed by CUNY IRB (File #2021 − 0567: The Impact of COVID-19 on New York’s Poorest: An Analysis of Excess Mortality During the Pandemic Using Burial Records from Hart Island) and does not involve human subjects as defined by CUNY’s Human Research Protection Program (HRPP).

### Excess mortality analysis

We estimate COVID-related excess mortality using unclaimed deaths (Hart Island burial records) that occurred during and prior to the initial COVID-19 outbreak. We provide estimates for the entire first year (epi-year 2020) and two key periods within it, namely, the six-month period from March 1 to August (epi-weeks 10–35) that marked the first wave of the pandemic in New York City (NYC) and the peak mortality week from Sunday April 5 to Saturday April 11 (epi-week 15). We note that COVID-19 deaths prior to epi-week 9 are unlikely. NYC reported its first infection on February 29, 2020. However, analysis of plasma samples of NYC Hospital patients suggests that the virus likely arrived during the week ending February 23^46^. As mentioned above, the first official death from COVID-19 in NYC occurred on March 11, 2020.

We define excess mortality in three ways. The difference between observed and expected (in the absence of COVID-19) death counts and death rates constitute the first two ways, and the ratio between observed and expected deaths counts makes up the third way. Positive values for excess counts or rates and ratios exceeding one (“1:1”) constitute *excess* mortality.

In our baseline or Approach 1 (A1) calculation of COVID-related excess death counts and rates, denoted by D_20,19_, the expected (counterfactual) death count and associated rate is based on observed unclaimed deaths in the comparable epidemiological period in 2019. Estimates of D_20,19_ for the three periods of interest (epi-year 2020, epi-weeks 10–35 and epi-week 15) are shown in Tables 2 and 3. (Since epi-year 2020 has 53 epi-weeks while 2019 (and 2018) only has 52 epi-weeks, the denominators in the underlying epi-year 2020 rate calculations are adjusted by a factor of 1 + 1/52 relative to the calendar year at-risk period.) Similarly, we provide evidence of excess mortality using ratios of unclaimed deaths, denoted by R_20/19_, using observed deaths in these periods.

Same-period prior-year comparisons have the benefit of reducing potential seasonal influences (e.g., all-cause mortality tends to be greater during winter months in temperate climate zones like New York^47,48^. However, a pre-pandemic trend in unclaimed deaths would bias the estimate of the 2020 counterfactual based on 2019 data. As shown in Table 1, unclaimed deaths increased by about 9% between 2018 and 2019, suggesting that it is necessary to adjust for trends. In Approach 2 (A2), we use the observed difference between 2018 and 2019 (D_19−18_) in the relevant epi-period to correct for possible (period-specific) linear pre-COVID trends. In Tables 2 and 3, we report the corresponding (linear) trend-corrected excess deaths counts and rates (“D^Adj^”=D_20−19_ -D_19−18_), and adjusted ratios (“R^Adj^” = deaths in 2020 / [deaths in 2019 + D^Count^_19−18_]).

In addition to calculating excess deaths based on counterfactuals obtained from the prior two years (A1 & A2), we also use predicted deaths from regression models estimated on data from the prior five years (A3), an empirical strategy found in many excess mortality analyses including those by the CDC^28^. Specifically, adapting methods developed in Heiland et al. (2026)^43^, we estimated count data regression models based on the epi-years 2015–2019 daily mortality data and used the fitted models to predict counterfactual unclaimed deaths counts for the three periods of interest in 2020 noted above. We report the corresponding excess counts and rates (“D^Reg^”), and ratios (“R^Reg^”) under A3 in Tables 2 and 3. An advantage of this approach to obtaining the counterfactuals is that it addresses potential confounding from seasonality and longer-term (non-linear) trends as it adjusts for time trends and seasonality. (Specification details and regression coefficients, along with model-level statistics, are provided in the Appendix.)

As a final comparison, and to place the excess mortality of unclaimed death in context, Table 4 uses all three approaches (A1-A3) with all deaths – claimed and unclaimed – in NYC.

Across approaches, we express excess deaths in terms of both rates and ratios. We use rates to illustrate absolute (excess) occurrence of unclaimed death – that is, how much more *frequent* than expected unclaimed deaths were in 2020 in the reference group (residential population) – and (excess) ratios to capture the percent occurrence – that is, how much more *likely* unclaimed deaths were in 2020 than expected. Both ways provide important and different insights; the rate perspective is particularly useful when comparing our results to other estimates of COVID-related excess death rates such as Lajous et al.^49^, whereas the ratio perspective is very helpful to illustrate impact magnitude of our results.

As reported in **Results**, the differences between the three approaches tend to be small and the results from A3, our most sophisticated approach to obtaining counterfactuals typically falls right between the results from simple comparison and extrapolation (A1 & A2). (We do not report ratios by borough given the smaller cell sizes. We do not standardize the excess mortality estimates by age: age-adjustments are used in excess mortality estimates when large samples or population-level data are available^30,50^, but given the nature of unclaimed mortality, this dataset is small. More importantly, given the short time period of this study, we are not concerned about changes in the population age structure that would necessitate age-standardization.)

## Results

Our analysis of the impact of the COVID-19 pandemic on mortality among disadvantaged New Yorkers based on Hart Island burial records examines timing and magnitude citywide, by borough and by sex. We focus on COVID-19 related excess deaths in 2020 overall and during the peak week (April 5 to 11, 2020) and the 6-month period (March 1 to August 29, 2020) considered the first wave in NYC. Magnitudes of excess death are inferred from relative unclaimed death count and rate comparisons across time (described in detail in **Methods**).

### Basic descriptives

As shown in Table 1, during epidemiological (herein epi-) year 2020, 2,529 deaths occurred to individuals who were buried on Hart Island (“unclaimed deaths”). This compares to 939 and 862 Hart Island deaths recorded in epi-year 2019 and 2018, respectively. In the 2018–2020 period, the average age at death was 68.6 years and almost three-quarters of unclaimed deaths in 2018–2020 were male (73.6%). Consistent with mortality patterns in the general population, the men buried on Hart Island were substantially younger than the women (average age: 67.3 men vs. 72.0 women). Also, consistent with known effects of the COVID-19 pandemic on mortality, the share of male unclaimed deaths was slightly higher (or the same as) in 2020 than in prior years (73.7% in 2020 vs. 73.3% in 2019 but 73.7% in 2018) and so was the average age at death (69.0 years in 2020 vs. 67.6 in 2019 and 68.4 in 2018), with similar increases for both sexes (66.4 in 2019 to 67.7 in 2020 for men vs. 70.9 to 72.4 for women). In 2020, 31.5% of all unclaimed deaths occurred in the Bronx, 24.7% in Brooklyn, 22.7% in Manhattan, 18.9% in Queens, and 2.1% on Staten Island. The share of deaths coming from Brooklyn was unusually high that year compared to earlier, consistent with Brooklyn being the most populous borough. (And notably, the relative share of unclaimed deaths was higher in 2018 in the Bronx than in other years, a pattern in the data that affects the results from A2 for the Bronx when compared to the results from other approaches, but not the overall findings.)

**Table 1:** Basic distribution and demographics of unclaimed deaths (Hart Island burials) and population sizes, NYC overall and by borough, 2018–2020.

		NYC	Borough				
Epi-year	Concept	Overall	The Bronx	Brooklyn	Queens	Manhattan	Staten Island
Unclaimed deaths (Hart Island burials)							
2020	Count	2,529	796	625	479	575	54
	%		31.5%	24.7%	18.9%	22.7%	2.1%
2019	Count	939	286	185	178	283	7
	%		30.5%	19.7%	19.0%	30.1%	0.7%
2018	Count	862	300	184	149	215	14
	%		34.8%	21.3%	17.3%	24.9%	1.6%
2018–2020	Count	4,330	1,382	994	806	1,073	75
	%		31.9%	23.0%	18.6%	24.8%	1.7%
Mean age at death							
2020	Years	69.0	68.5	68.4	69.4	69.8	67.9
2019	Years	67.6	67.9	67.8	67.7	67.1	69.5
2018	Years	68.4	67.9	67.0	67.6	71.0	67.4
2018–2020	Years	68.6	68.3	68.1	68.7	69.3	68.0
Percent male							
2020	%	73.7%	73.7%	74.1%	74.7%	72.2%	77.8%
2019	%	73.3%	75.5%	70.1%	74.0%	72.8%	71.4%
2018	%	73.7%	73.7%	77.3%	70.7%	71.4%	92.9%
2018–2020	%	73.6%	74.1%	73.9%	73.8%	72.2%	80.0%
Total population							
2020	Years	8,740,647	1,461,125	2,719,044	2,388,586	1,677,306	494,586
2019	Years	8,336,817	1,418,207	2,559,903	2,253,858	1,628,706	476,143
2018	Years	8,390,081	1,432,087	2,578,074	2,274,605	1,629,055	476,260
Male population							
2020	Years	4,209,227	692,685	1,299,427	1,170,548	803,915	242,652
2019	Years	3,978,439	669,548	1,212,194	1,093,889	771,478	231,330
2018	Years	4,003,443	675,622	1,221,692	1,103,811	771,374	230,944

Epi-year 2020 has 53 epi-weeks while 2019 and 2018 only have 52 epi-weeks. Population statistics are mid-year estimates.

### Citywide temporal dynamics

**Fig. 1:**
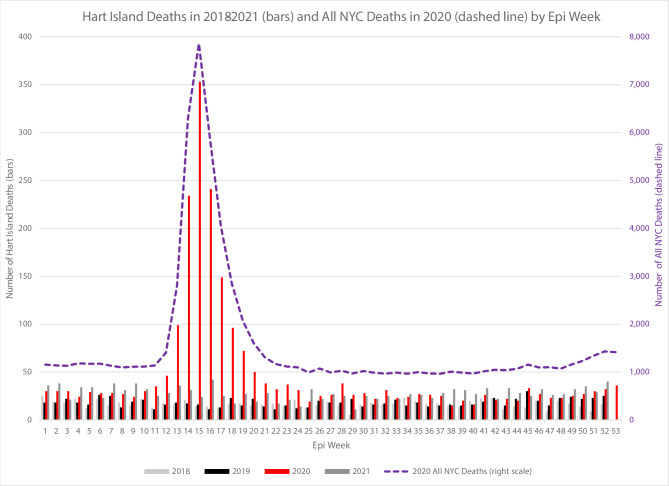
Weekly death counts in New York City, unclaimed deaths in 2018–2021 and all deaths in 2020.

As shown in Fig. 1, unclaimed deaths started to dramatically deviate from their prior years’ pattern in the second week of March 2020 and reached a sharp peak during epi-week 15 (Sunday April 5th to Saturday April 11th), followed by a rapid decline in deaths from mid-April to end of June. Figure 1 also includes two important points of reference: (1) the dashed line shows city-wide death counts, which displays identical timing patterns to unclaimed deaths in terms of onset, peak and decline of mortality and (2) unclaimed deaths for 2021, which – while still elevated relative to pre-2020 – are notably more similar in their distribution to the pre-pandemic years. Table 2 reports unclaimed deaths counts and rates during this peak week, the 6-month period (March 1 to August 29, 2020) considered the first wave in NYC, and for 2020 overall. It also shows corresponding estimates of excess unclaimed mortality based on differences in deaths (D, D^Adj^, & D^Reg^) and ratios of deaths (R, R^Adj^, & R^Reg^) for the City overall and by sex (see **Methods**). Rates are calculated using unclaimed deaths relative to the residential population and expressed in per 100,000.

Overall, there were 28.4 unclaimed deaths per 100,000 New Yorkers in 2020 (Table 2, data columns 1–2). Following our baseline approach (A1), i.e., comparing 2020 to 2019, we estimate a COVID-related excess death rate in 2020 of 17.1 per 100,000 New Yorkers (95% CI: 15.8–18.5). Adjusting for the pre-2020 linear trend (rows indicated as D^Adj^), the excess death rate is 16.1 (CI: 14.2–18.1) per 100,000 (A2). In the model-based approach using 5 years prior (A3), the excess death rate is 17.3 (CI: 15.9–18.6) per 100,000.

For the 6-month period from March to August, the (annualized) excess mortality rate is almost twice the 2020 overall excess rate at 31.7 (95% CI: 29.5–33.8) according to our baseline approach (A2: 31.6 [CI: 28.6–34.5]; A3: 31.5 [29.3–33.6]) per 100,000, consistent with a devastating impact of the COVID-19 pandemic during the initial outbreak in the City. During the peak in April, the weekly excess death rate reached 200 (95% CI: 177.5-222.6) (A2: 198.7 [CI: 174.2-223.2]; A3: 199.6 [CI: 177.1-222.2]) per 100,000, suggesting that 200 additional unclaimed deaths occurred for every 100,000 residents.

As shown in lower third of Table 2, at the peak of weekly deaths there were 22.1 (95% CI: 11.1–33.0) deaths in 2020 for every death in 2019 (A1: R_20/19_, “Overall” column, third-from-bottom row). Adjusting for the increase in unclaimed deaths observed during the same epi-week in 2018–2019 (capturing a pre-2020 linear trend), the death ratio still is a staggering 19.6:1 (CI: 10.4–28.8) (A2: R^Adj^, penultimate bottom row). Our regression-based approach yields a similar death ratio of 20.3 (A3: R^Reg^, bottom row), suggesting that about 20 times (CI: 10.5–30.1) as many unclaimed deaths occurred during the peak period than would have been expected in the absence of COVID-19.

The unclaimed death ratio (R) during the 6-month period generally considered the first wave of the pandemic in NYC (March 1 to August 29, 2020; epi-weeks 10 to 35) is 4.3 to 1 (95% CI: 3.7–4.8) in the baseline calculation (A1), same under linear-trend adjustment (A2), and slightly lower at 4.0 (CI: 3.6–4.4) under regression-based adjustment (A3), suggesting a COVID-related 300 to 330% increase in unclaimed mortality. For the entire first year of the pandemic, the ratios are 2.5 (CI: 2.3–2.7) (A2) and 2.6 (2.4–2.8) (A3) based on the adjusted approaches (A1: 2.7 [CI: 2.5–2.9]), implying COVID-related mortality increases in the range of 150 to 170%, respectively. The comparison across the three periods shows how concentrated (excess) unclaimed mortality was in 2020 and how closely its dynamics aligns with the known timing of the COVID-19 outbreak in the City.

Subgroup analyses by sex (Table 2, data columns 3-6) reveals higher COVID-related excess death *rates* for men than women but similar death *ratios*. The latter reflects the relatively small number of female unclaimed deaths in 2019, which magnifies ratio-based comparisons. In rate terms, emphasizing the absolute number of deaths, there were two to three times more COVID-related excess unclaimed deaths among men than women. For example, for 2020 overall there were 26.2 (95% CI: 23.9-28.6) (A1) (A2: 24.7 [CI: 21.1-28.2]; A3: 26.6 [24.3-29.0]) additional unclaimed male deaths per 100,000 male residents compared to 8.6 (CI: 7.3-10.0) (A1) (A2: 8.0 [CI: 6.1-10.0]; A3: 8.7 [7.3-10.1]) per 100,000 among women. In the March-August period, we find that there were 48.4 (CI: 44.5-52.3) (A1) (A2: 47.8 [CI: 42.6-53.1]; A3: 48.9 [44.8-52.7]) additional unclaimed deaths per 100,000 residents among men compared to 16.1 (CI: 13.9-18.3) (A1) (A2: 16.3 [CI: 13.6-19.0]; A3: 15.6 [13.4-17.7]) per 100,000 among women. Looking at the mortality ratio (R), for 2020 overall there were 2.7 (CI: 2.5-2.9) (A1) (A2: 2.5 [CI: 2.3-2.7]; A3: 2.6 [2.4-2.8]) times the expected number of male deaths compared to 2.7 (CI: 2.3-3.0) (A1) (A2: 2.4 [CI: 2.0-2.8]; A3: 2.6 [2.2-3.0]) times among women. The corresponding March-August values are also very similar across the sexes: 4.3 [CI: 3.7-4.8] (A1) (A2 & A3: 4.1 [CIs: 3.5-4.7]) for males vs. 4.4 (3.5-5.4) (A1) (A2: 4.7 [CI: 3.7-5.6]; A3: 3.9 [3.1-4.7]) for females. To understand age at death during this crucial initial wave of the COVID-19 outbreak, Box 1 provides additional evidence comparing unclaimed deaths to confirmed COVID-19 deaths within the Mount Sinai Hospital System (MSHS).

**Table 2:** Unclaimed deaths (counts & rates), excess unclaimed deaths (counts & rates), and unclaimed death ratios, NYC overall and by sex, 2018–2020.

Concept	Period	Overall		Males		Females	
		Counts	Rates	Counts	Rates	Counts	Rates
Unclaimed deaths (observed)							
2020	Epi-year	2,529	28.4	1,865	43.5	664	14.4
2019	Epi-year	939	11.3	687	17.3	250	5.7
2018	Epi-year	862	10.3	630	15.7	225	5.1
Excess unclaimed deaths							
A1: D_20−19_=2020 − 2019	Epi-year	1,590	17.1	1,178	26.2	414	8.6
A2: D^Adj^_20−19_=D_20−19_-D_19−18_	Epi-year	1,513	16.1	1,121	24.7	389	8.0
For A2: D_19−18_=2019 − 2018	Epi-year	77	1.0	57	1.5	25	0.6
A3: D^Reg^=2020 Obs.-2020 Pred.	Epi-year	1,558	17.3	1,156	26.6	406	8.7
For A3: 2020 predicted from regression	Epi-year	971	11.1	709	16.8	258	5.7
A1: R_20/19_=2020/2019	Epi-year	2.7		2.7		2.7	
A2: R^Adj^_20/19_=2020/(2019 + D_19−18_)	Epi-year	2.5		2.5		2.4	
A3: R^Reg^=2020 Obs./2020 Pred.	Epi-year	2.6		2.6		2.6	
Unclaimed deaths (observed)							
2020	Epi-week 10 to 35	1,832	41.9	1,356	64.4	476	21.0
2019	Epi-week 10 to 35	427	10.2	319	16.0	107	4.9
2018	Epi-week 10 to 35	426	10.2	310	15.5	112	5.1
Excess unclaimed deaths							
A1: D_20−19_=2020 − 2019	Epi-week 10 to 35	1,405	31.7	1,037	48.4	369	16.1
A2: D^Adj^_20−19_=D_20−19_-D_19−18_	Epi-week 10 to 35	1,404	31.6	1,028	47.8	374	16.3
For A2: D_19−18_=2019 − 2018	Epi-week 10 to 35	1	0.1	9	0.5	-5	-0.2
A3: D^Reg^=2020 Obs.-2020 Pred.	Epi-week 10 to 35	1,376	31.5	1,026	48.8	353	15.6
For A3: 2020 predicted from regression	Epi-week 10 to 35	456	10.4	330	15.7	123	5.4
A1: R_20/19_=2020/2019	Epi-week 10 to 35	4.3		4.3		4.4	
A2: R^Adj^_20/19_=2020/(2019 + D_19−18_)	Epi-week 10 to 35	4.3		4.1		4.7	
A3: R^Reg^=2020 Obs./2020 Pred.	Epi-week 10 to 35	4.0		4.1		3.9	
Unclaimed deaths (observed)							
2020	Epi-week 15 (Peak)	353	210.0	262	323.7	91	104.4
2019	Epi-week 15	16	10.0	12	15.7	4	4.8
2018	Epi-week 15	14	8.7	11	14.3	3	3.6
Excess unclaimed deaths							
A1: D_20−19_=2020 − 2019	Epi-week 15 (Peak)	337	200.0	250	308.0	87	99.7
A2: D^Adj^_20−19_=D_20−19_-D_19−18_	Epi-week 15 (Peak)	335	198.7	249	306.6	86	98.4
For A2: D_19−18_=2019 − 2018	Epi-week 15	2	1.3	1	1.4	1	1.2
A3: D^Reg^=2020 Obs.-2020 Pred.	Epi-week 15 (Peak)	336	199.6	249	307.8	86	98.9
For A3: 2020 predicted from regression	Epi-week 15 (Peak)	17	10.4	13	15.9	5	5.6
A1: R_20/19_=2020/2019	Epi-week 15 (Peak)	22.1		21.8		22.8	
A2: R^Adj^_20/19_=2020/(2019 + D_19−18_)	Epi-week 15 (Peak)	19.6		20.2		18.2	
A3: R^Reg^=2020 Obs./2020 Pred.	Epi-week 15 (Peak)	20.3		20.4		20.2	

D_t, t−1_=Difference in death counts or rates (t minus t-1). Approach 1 (A1): D_20−19_=2020 − 2019. Approach 2 (A2): D^Adj^_20−19_=Linear-trend adjusted difference in deaths counts or rates (D_20−19_-D_19−18_). Approach 3 (A3): D^Reg^=2020 Observed − 2020 Regression-Predicted. Rates are per 100,000 Residents. A1: R_20/19_=Ratio of 2020 over 2019 observed death counts. A2: R^Adj^_20/19_=Linear-trend adjusted ratio (2020 observed over 2019 observed plus D_19−18_). A3: R^Reg^=Regression-adjusted ratio (2020 observed over 2020 regression-predicted). The fitted model underlying A3 is shown in Appendix Table A1. Since epi-year 2020 (a leap year) has 53 epi-weeks while 2019 and 2018 only have 52 epi-weeks, the denominators in epi-year 2020 rate calculations are adjusted by a factor 1 + 1/52. Epi-week 10 to 35 in 2020 is March 1 to August 29. Epi-week 15 in 2020 is Sunday April 5 to Saturday April 11, the week unclaimed deaths peaked.

**Box 1**. Comparing demographic profiles of unclaimed deaths to COVID-19 deaths in the Mt. Sinai Hospital System (MSHS) in New York City (NYC). Because the Hart Island records include information on place of death (address and name, in the case of facilities), they present an opportunity to directly compare age and sex demographics of unclaimed deaths to confirmed COVID-19 deaths that occurred at hospitals within the Mount Sinai Health System (MSHS) using results from a study by Klang and colleagues^39^. MSHS is located throughout NYC.

While caution is in order given the limited number of cases and lack of cause-of-death information in the Hart Island records, the younger ages at death among these hospital deaths during the first wave of the pandemic are consistent with the idea that individuals, especially women, who die unclaimed are selected from a population that is more physically vulnerable at any age (e.g., more likely to suffer from chronic conditions) than New Yorkers overall.

### Findings from borough-level analysis

**Fig. 2:**
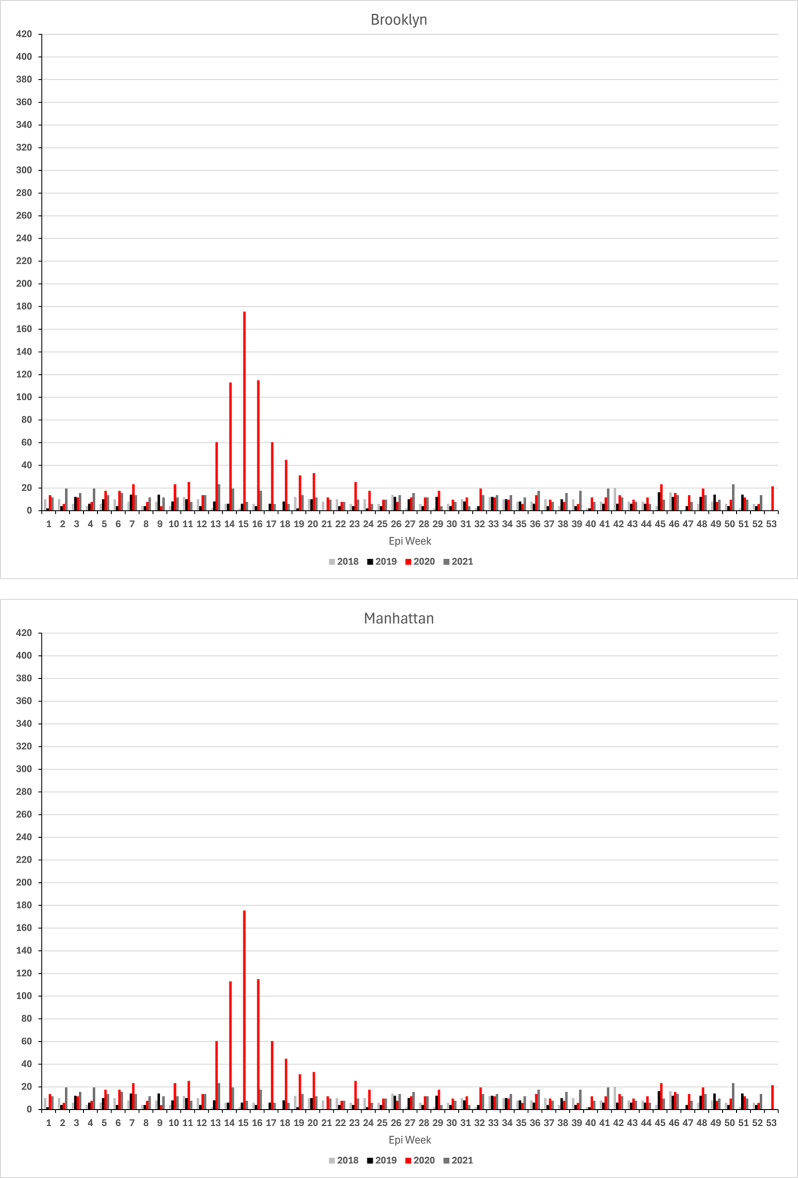

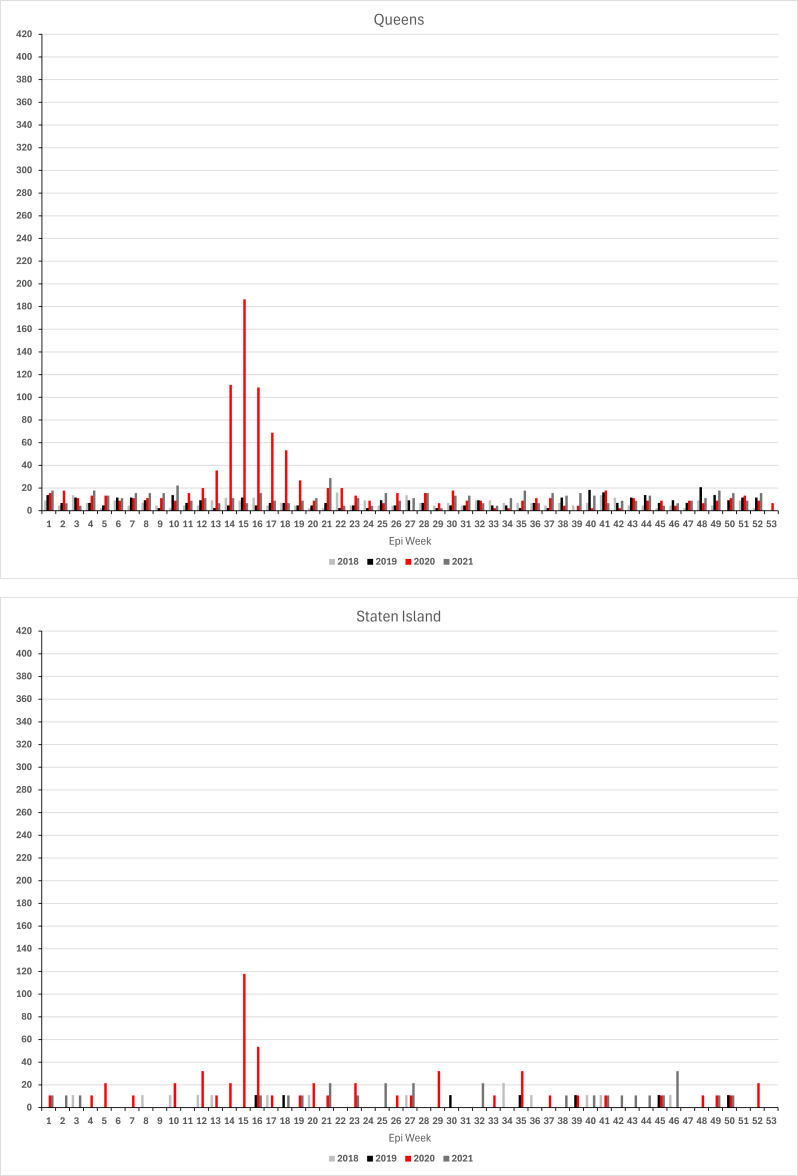

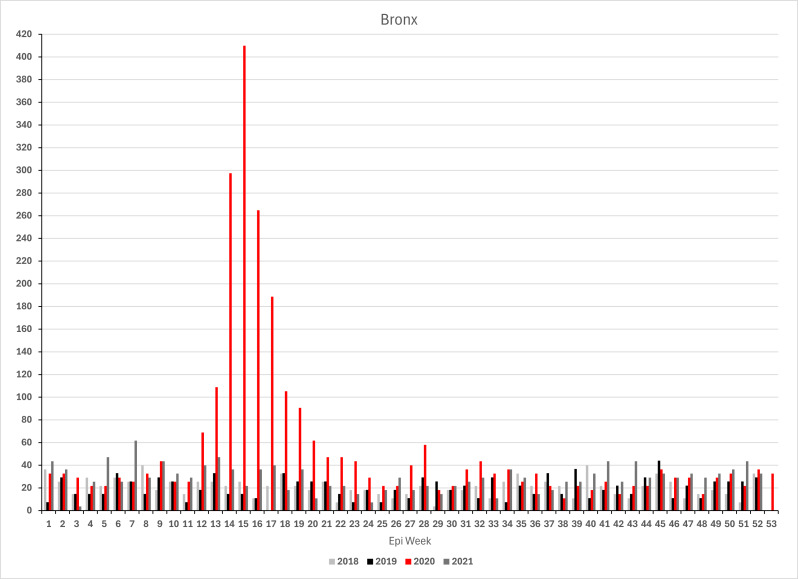
Weekly unclaimed death rates (deaths per 100,000 residents) by borough, 2018 to 2020.

Borough-level analyses of the location at death of the individuals buried on Hart Island show great similarity in the timing of how the 2020 outbreak affected vulnerable New Yorkers across the City but also revealed substantial variation in the magnitude of the impact. As shown in Fig. 2, (annualized) weekly deaths rates in 2020 peaked in the same week (epi-week 15) in all five boroughs. The death rate increased first in the Bronx in epi-week 12 (ending Saturday, March 23). A week later, rates start to take off in Brooklyn, Manhattan, and Queens. On Staten Island, a pronounced increase is not observed until the peak in epi-week 15. Across boroughs, rates declined after that and, by the beginning of summer, reached levels seen during the first two months of the same year. Compared to 2018 and 2019, death rates remained elevated throughout 2020 in the Bronx, Manhattan and on Staten Island, whereas rates in Queens approached levels only slightly above those seen in prior years. Evidence that the Bronx was the first borough to see the steep climb in Hart Island mortality rate (dramatically deviating from the prior year pattern) is noteworthy given questions about whether the initial spread of COVID-19 in NYC occurred in Queens or in the Bronx (see, e.g., Dellicour et al.^51^).

At their peak, the Hart Island weekly (annualized) excess death rate reached almost 400 deaths (i.e., A1: 387.5 [95% CI: 312.0-462.9]; A2: 398.2 [316.5–480.0]; A3: 382.1 [304.6-459.5]) per 100,000 residents in the Bronx (see Fig. 2; Table 3)—almost double the citywide excess rate (A1: 200 [177.5-222.6] per 100,000; A2: 198.7 [174.2-223.2]; A3: 199.6 [177.1-222.2]) and more than twice the next highest rate of 171.3 [CI: 131.0-211.7] (A1) (A2: 168.9 [124.1-213.8]; A3: 175.4 [132.7-218.2]) deaths per 100,000 in Queens. The corresponding rates in Brooklyn and Manhattan were 166 (CI: 129.8-202.3) (A1) (A2: 161.9 [123.5-200.4]; A3: 165.7 [127.4-203.7]) and 157.7 (CI: 110.9-204.6) (A1) (A2: 151.4 [99.0-203.7]; A3: 155.4 [108.6-202.3]), respectively. On Staten Island, the weekly excess rate peaked at 115.7 deaths (CI: 47.2-184.1) (A1 & A2) (A3: 113.8 [30.0-197.7]) per 100,000.

**Table 3:** Unclaimed death and excess unclaimed death rates (per 100,000 residents), NYC overall and by borough, 2018–2020.

Concept	Period	NYC	Borough				
		Overall	The Bronx	Brooklyn	Queens	Manhattan	Staten Island
Unclaimed deaths (observed)			Hart Island deaths per 100,000 residents				
2020	Epi-year	28.4	53.5	22.6	19.7	33.6	10.7
2019	Epi-year	11.3	20.2	7.2	7.9	17.4	1.5
2018	Epi-year	10.3	20.9	7.1	6.6	13.2	2.9
Excess unclaimed deaths							
A1: D_20−19_=2020 − 2019	Epi-year	17.1	33.3	15.3	11.8	16.3	9.2
A2: D^Adj^_20−19_=D_20−19_-D_19−18_	Epi-year	16.1	34.1	15.2	10.4	12.1	10.7
For A2: D_19−18_=2019 − 2018	Epi-year	1.0	-0.8	0.1	1.3	4.2	-1.5
A3: D^Reg^=2020 Obs.-2020 Pred.	Epi-year	17.3	31.9	15.6	11.7	17.4	8.8
For A3: 2020 pred. from reg.	Epi-year	11.1	21.5	6.9	8.0	16.2	1.9
Unclaimed deaths (observed)							
2020	Epi-week 10–35	41.9	81.9	33.4	30.1	45.3	16.2
2019	Epi-week 10–35	10.2	18.3	6.6	5.9	17.3	1.7
2018	Epi-week 10–35	10.2	19.4	6.8	6.9	13.8	3.4
Excess unclaimed deaths							
A1: D_20−19_=2020 − 2019	Epi-week 10–35	31.7	63.5	26.8	24.2	28.0	14.5
A2: D^Adj^_20−19_=D_20−19_-D_19−18_	Epi-week 10–35	31.6	64.6	26.9	25.2	24.4	16.2
For A2: D_19−18_=2019 − 2018	Epi-week 10–35	0.1	-1.1	-0.2	-1.0	3.6	-1.7
A3: D^Reg^=2020 Obs.-2020 Pred.	Epi-year	31.5	61.6	26.9	22.7	30.1	14.4
For A3: 2020 pred. from reg.	Epi-year	10.4	20.2	6.5	7.5	15.2	1.8
Unclaimed deaths (observed)							
2020	Epi-week 15 (Peak)	210.0	402.2	172.1	182.9	170.5	115.7
2019	Epi-week 15	10.0	14.7	6.1	11.5	12.8	0.0
2018	Epi-week 15	8.7	25.4	2.0	9.1	6.4	0.0
Excess unclaimed deaths							
A1: D_20−19_=2020 − 2019	Epi-week 15 (Peak)	200.0	387.5	166.0	171.3	157.7	115.7
A2: D^Adj^_20−19_=D_20−19_-D_19−18_	Epi-week 15 (Peak)	198.7	398.2	161.9	168.9	151.4	115.7
For A2: D_19−18_=2019 − 2018	Epi-week 15	1.3	-10.8	4.1	2.4	6.4	0.0
A3: D^Reg^=2020 Obs.-2020 Pred.	Epi-week 15 (Peak)	199.6	382.1	165.7	175.4	155.4	113.8
For A3: 2020 pred. from reg.	Epi-week 15 (Peak)	10.4	20.1	6.5	7.4	15.1	1.8

D_t, t−1_=Difference in death counts or rates (t minus t-1). Approach 1 (A1): D_20−19_=2020 − 2019. Approach 2 (A2): D^Adj^_20−19_=Linear-trend adjusted difference in deaths counts or rates (D_20−19_-D_19−18_). Approach 3 (A3): D^Reg^=2020 Observed − 2020 Regression-Predicted. The fitted model underlying A3 is shown in Appendix Table A2. Rates are per 100,000 Residents. Since epi-year 2020 (a leap year) has 53 epi-weeks while 2019 and 2018 only have 52 epi-weeks, the denominators in epi-year 2020 rate calculations are adjusted by a factor 1 + 1/52. Epi-week 10 to 35 in 2020 is March 1 to August 29. Epi-week 15 in 2020 is Sunday April 5 to Saturday April 11, the week unclaimed deaths peaked.

### Excess mortality: unclaimed deaths on Hart Island versus all deaths in NYC

Table 4 replicates the excess mortality analysis in Table 2 using data on all NYC deaths. This places our results for the unclaimed in a broader context of city-wide excess mortality. In terms of mortality onset and peak, Hart Island follows the City perfectly but – across all approaches (A1-A3) – excess death ratios are orders of magnitude greater among unclaimed deaths. Based on all deaths in 2020, the excess death ratio (R) is approximately 1.5 to 1 across all approaches (Table 4; A1: 1.53 [CI: 1.51–1.55]; A2: 1.54 [1.52–1.56]; A3: 1.49 [1.47–1.51]), compared to 2.5–2.7 to 1 for unclaimed deaths only (Table 2; A1: 2.69 [CI: 2.50–2.89]; A2: 2.49 [2.31–2.67]; A3: 2.60 [2.41–2.80]). In the six months spanning the initial outbreak, the excess death ratio is approximately 2 to 1 based on all deaths across approaches (A1: 2.01 [CI: 1.97–2.05]; A2: 2.00 [1.96–2.04]; A3: 1.99 [1.95–2.03]), while it is more than twice that at 4-4.3 to 1 for unclaimed deaths (A1: 4.29 [CI: 3.84–4.74]; A2: 4.28 [3.83–4.73]; A3: 4.01 [3.60–4.43]). During the peak week of the pandemic, the ratios are 7.5–7.7 for all deaths (A1: 7.46 [CI: 6.99–7.93]; A2: 7.75 [7.24–8.26]; A3: 7.56 [7.07–8.05]), compared to 19.6–22.1 for Hart Island deaths (A1: 22.06 [CI: 11.01–33.12]; A2: 19.61 [10.32–28.90]; A3: 20.26 [10.52–30.01]), implying almost three times greater COVID-related excess mortality among those unclaimed compared to all deaths. By sex, we note that excess mortality based on all deaths is consistently greater for men than women, while it is generally the same or greater among women based on unclaimed deaths.

**Table 4:** All deaths (counts & rates), excess deaths (counts & rates), and death ratios, NYC overall and by sex, 2018–2020.

Concept	Period	Overall		Males		Females	
		Counts	Rates	Counts	Rates	Counts	Rates
Deaths (observed)							
2020	Epi-year	83,074	932.5	44,102	1,028.0	38,972	843.8
2019	Epi-year	54,390	652.4	27,555	692.6	26,835	615.7
2018	Epi-year	54,905	654.4	27,328	682.6	27,577	628.7
Excess deaths							
A1: D_20−19_=2020 − 2019	Epi-year	28,684	280.1	16,547	335.4	12,137	228.1
A2: D^Adj^_20−19_=D_20−19_-D_19−18_	Epi-year	29,199	282.1	16,320	325.4	12,879	241.1
For A2: D_19−18_=2019 − 2018	Epi-year	-515	-2.0	227	10.0	-742	-12.9
A3: D^Reg^=2020 Obs.-2020 Pred.	Epi-year	27,238	293.7	15,771	354.9	11,462	236.7
For A3: 2020 predicted from regression	Epi-year	55,836	638.8	28,331	673.1	27,510	607.1
A1: R_20/19_=2020/2019	Epi-year	1.5		1.6		1.5	
A2: R^Adj^_20/19_=2020/(2019 + D_19−18_)	Epi-year	1.5		1.6		1.5	
A3: R^Reg^=2020 Obs./2020 Pred.	Epi-year	1.5		1.6		1.4	
Deaths (observed)							
2020	Epi-week 10 to 35	52,736	1,206.7	28,574	1,357.7	24,162	1,066.4
2019	Epi-week 10 to 35	26,200	628.5	13,387	673.0	12,813	588.0
2018	Epi-week 10 to 35	26,037	620.7	13,085	653.7	12,952	590.5
Excess deaths							
A1: D_20−19_=2020 − 2019	Epi-week 10 to 35	26,536	578.1	15,187	684.7	11,349	478.4
A2: D^Adj^_20−19_=D_20−19_-D_19−18_	Epi-week 10 to 35	26,373	570.3	14,885	665.4	11,488	481.0
For A2: D_19−18_=2019 − 2018	Epi-week 10 to 35	163	7.9	302	19.3	-139	-2.5
A3: D^Reg^=2020 Obs.-2020 Pred.	Epi-week 10 to 35	26,268	601.1	15,043	714.7	11,220	495.2
For A3: 2020 predicted from regression	Epi-week 10 to 35	26,468	605.6	13,531	642.9	12,942	571.2
A1: R_20/19_=2020/2019	Epi-week 10 to 35	2.0		2.1		1.9	
A2: R^Adj^_20/19_=2020/(2019 + D_19−18_)	Epi-week 10 to 35	2.0		2.1		1.9	
A3: R^Reg^=2020 Obs./2020 Pred.	Epi-week 10 to 35	2.0		2.1		1.9	
Deaths (observed)							
2020	Epi-week 15 (Peak)	7,862	4,677.3	4,423	5,464.1	3,439	3,946.4
2019	Epi-week 15	1,054	657.4	541	707.1	513	612.1
2018	Epi-week 15	1,093	677.4	506	657.2	587	695.8
Excess deaths							
A1: D_20−19_=2020 − 2019	Epi-week 15 (Peak)	6,808	4,019.9	3,882	4,757.0	2,926	3,334.3
A2: D^Adj^_20−19_=D_20−19_-D_19−18_	Epi-week 15 (Peak)	6,847	4,039.9	3,847	4,707.1	3,000	3,418.1
For A2: D_19−18_=2019 − 2018	Epi-week 15	-39	-20.0	35	49.9	-74	-83.8
A3: D^Reg^=2020 Obs.-2020 Pred.	Epi-week 15 (Peak)	6,821	4,058.2	3,891	4,807.2	2,930	3,317.5
For A3: 2020 predicted from regression	Epi-week 15 (Peak)	1,041	619.1	532	656.9	509	628.9
A1: R_20/19_=2020/2019	Epi-week 15 (Peak)	7.5		8.2		6.7	
A2: R^Adj^_20/19_=2020/(2019 + D_19−18_)	Epi-week 15 (Peak)	7.7		7.7		7.8	
A3: R^Reg^=2020 Obs./2020 Pred.	Epi-week 15 (Peak)	7.6		8.3		6.8	

D_t, t−1_=Difference in death counts or rates (t minus t-1). Approach 1 (A1): D_20−19_=2020 − 2019. Approach 2 (A2): D^Adj^_20−19_=Linear-trend adjusted difference in deaths counts or rates (D_20−19_-D_19−18_). Approach 3 (A3): D^Reg^=2020 Observed − 2020 Regression-Predicted. Rates are per 100,000 Residents. A1: R_20/19_=Ratio of 2020 over 2019 observed death counts. A2: R^Adj^_20/19_=Linear-trend adjusted ratio (2020 observed over 2019 observed plus D_19−18_). A3: R^Reg^=Regression-adjusted ratio (2020 observed over 2020 regression-predicted). The fitted model underlying A3 is shown in Appendix Table A3. Since epi-year 2020 (a leap year) has 53 epi-weeks while 2019 and 2018 only have 52 epi-weeks, the denominators in epi-year 2020 rate calculations are adjusted by a factor 1 + 1/52. Epi-week 10 to 35 in 2020 is March 1 to August 29. Epi-week 15 in 2020 is Sunday April 5 to Saturday April 11, the week unclaimed deaths peaked.

## Discussion

This paper provides new evidence on the temporal and spatial impact of the COVID-19 outbreak on mortality in New York City (NYC) using burial records from Hart Island—the City’s public cemetery. Among this population of highly vulnerable New Yorkers, there is strong evidence of dramatic excess deaths due to COVID-19 in 2020. Hart Island deaths started to deviate from their expected historical pattern in the second week of March and reached a peak during the week ending April 11, 2020. At the peak of weekly deaths during the first wave of the outbreak in NYC (epidemiological week 15 in 2020), 20 to 22 times as many unclaimed deaths occurred than expected in the absence of the pandemic. During the 6-month period of the first wave of COVID-19 in NYC (March 1 to August 29, 2020), we observe 4.0 to 4.3 times as many unclaimed deaths than expected, and for 2020 overall, we find that this figure is still a remarkable 2.5 to 2.7. Across boroughs excess unclaimed deaths counts and rates peaked during the exact same week, but rates differed greatly across the City with excess unclaimed death rates more than twice as high in the Bronx than in the other counties.

### Hart Island deaths in the context of early pandemic impact

It is important to understand our results based on Hart Island deaths in the greater context of previous evidence from NYC (and beyond). We have shown unambiguously that the temporal distribution of unclaimed deaths matches the pattern of COVID-related deaths for the City overall. Confirmed and suspected deaths for NYC were heavily concentrated over a period of just seven weeks between the end of March and mid-May of 2020: The highest COVID-19 death toll in the City was observed on April 7 with 597 confirmed and 216 probable COVID-19 deaths.^20^ We find that the single most deadly day based on Hart Island burials was April 8, 2020, with 57 deaths (and compared to 4 deaths the same Wednesday in the year prior). Nationwide, during the first wave, the number of confirmed COVID-19 deaths reached a high of 2,297 on April 6, 2020.^52^.

However, excess all-cause deaths (ratios) based on Hart Island are substantially greater than the official share of COVID-19 deaths and comparable existing estimates for the City overall, reinforcing how aggregate statistics can mask important heterogeneity and vulnerabilities in the population.^53^ Based on CDC weekly deaths, in the 12-week period between February 22 and May 15 in 2020, there were approximately 1.5 deaths per death in comparable periods in 2017–2019 in NYC.^52^ This compares to almost 7.2 Hart Island deaths in 2020 for every Hart Island death in 2019 in the comparable period (our calculation). Böttcher et al.^54^ estimate that at their peak, average weekly deaths from all causes in NYC were about eight times greater than in previous years. This pales in comparison to our estimate of 20:1 to 22:1 based on peak weekly unclaimed deaths (epidemiological week 15). Lajous et al.^49^ estimate a citywide COVID-related annualized excess death rate of 311 per 100,000 NYers for March to August 2020. For this period, we estimate an (annualized) Hart Island excess death rate of 31 per 100,000 New Yorkers, which suggests that about 10% of all COVID-related excess deaths that occurred between March and August 2020 were interred on Hart Island. To put this in context, we estimate that in 2019, 1.7% of all deaths in NYC were unclaimed, and in 2020, this figure rose to 3%.

Böttcher et al.^54^ (and others) interpret the fact that their estimates of excess deaths during the initial outbreak greatly exceeded official COVID-19 deaths as evidence that official figures substantially undercount deaths from COVID-19 (reflecting lack of testing and generally poor surveillance). Given the close temporal match between the Hart Island data and the citywide timing of COVID-19 spread during the initial outbreak, it is possible that the estimated excess for this period is largely accounted for by deaths directly related to COVID-19 illness. However, increases in several major causes of death other than COVID-19 are very likely to have contributed to the observed excess unclaimed deaths as well. Supporting this claim, NYC Department of Health and Mental Hygiene (Table C3, p.61)^1^ data show substantial increases in other causes of deaths between 2019 and 2020: diseases of the heart (19.3%), heart failure (cerebrovascular disease: 16.1%, essential hypertension and renal disease: 20%), psychoactive substance use and accidental drug poisoning (39.5%), diabetes mellitus (17.2%), and influenza and pneumonia (26.2%)^55^. The rise in the official influenza and pneumonia mortality is especially notable in light of earlier findings of significant undercounts in influenza and pneumonia deaths in New York state during the first three months of the pandemic.^56^ Deaths from influenza and pneumonia, diabetes and cerebrovascular disease, as well as deaths from external causes increased noticeably more in the Bronx, Brooklyn, and Queens than in the more affluent Manhattan.^55^ Taken together, the dramatically greater excess mortality based on Hart Island records compared to the City overall points to a severe impact of COVID-19 on New York’s most vulnerable populations both directly, via greater mortality from COVID-19 illness, and indirectly, via greater mortality from chronic health conditions and behaviors exacerbated during the outbreak (also see Box 1).

### Social vulnerability and unclaimed death in context

While there is little systematic evidence on the characteristics of those whose bodies are unclaimed upon death, a study of unclaimed deaths in Los Angeles found area-level unemployment, poverty, and Black minority status were important predictors of unclaimed death rates.^15,16^ Evidence from unclaimed deaths in Indianapolis showed decedents were disproportionately male and younger at death.^17^ In our analysis of sex and age-at-death patterns in the Hart Island data (see Table 1), we see the same demographic skews. We also add to this emerging literature by documenting that this gender imbalance was exacerbated by the COVID-19 pandemic, as evidenced by higher excess mortality rates among men than women, consistent with the (globally) well-documented pattern that men face greater risk of mortality during the COVID-19 pandemic than women.^57,58^.

We find that Hart Island COVID-related excess death rates (adjusted for population) in 2020 were greatest in the Bronx and lowest on Staten Island, with Brooklyn, Queens, and Manhattan in between. This is consistent with the hypothesis that economic vulnerability plays a key role in becoming an unclaimed death^16,18,59^: Greater poverty may increase the rate of unclaimed mortality through multiple channels: (i) by increasing all-cause (and all-burial-type) mortality, (ii) by increasing the likelihood that a death will remain unclaimed as a result of fewer economic resources to afford a private funeral (direct moderating effect) and due to greater social isolation (indirect moderating effect through social isolation), and (iii) by worsening the impact of external shocks such as the COVID-19 pandemic on mortality (especially among the poor).

Empirically, there are notable differences in household income, population in poverty, and population under age 65 without health insurance across the five boroughs of New York City. In particular, Manhattan and Staten Island have higher median household income levels and lower rates of poverty and uninsured. According to 2017–2021 ACS 5-year estimates from the U.S. Census Bureau, the median household income (in 2021 dollars) in Manhattan was $93,956, 17.4% of the population were considered living in poverty and 4.7% of those under 65 lacked health insurance. In contrast, the median household income (% in poverty, % uninsured) in the Bronx was $43,726 (26.4%, 8.8%), in Brooklyn it was $67,753 (19.2%, 7.6%), in Queens it was $75,886 (13.6%, 9.7%), and on Staten Island it was $89,427 (11.9%, 4.4%)^60^.

Boroughs (and neighborhoods within them) likely differ in other important aspects as well and those factors could counteract the effects of economic disparities. On the one hand, there is variation in age, for example, that may benefit less affluent parts of the City in terms of COVID-19-related mortality. Specifically, the 2017–2021 ACS share of persons 65 and older is lowest in the Bronx (14.0%) and Brooklyn (15.1%) and highest in Manhattan (18.3%), with Queens (17.4%) and Staten Island (17.0%) falling somewhere in between. Another socio-demographic risk factor for COVID-19 is persons per household (proxying for density/risk of spread). Queens and Staten Island stand out in this aspect with 2.93 and 2.87 persons per household according to the 2017–2021 ACS, compared to 2.73, 2.71, and 2.09 in the Bronx, Brooklyn, and Manhattan, respectively.^60^ On the other hand, fewer persons per household may also be an indicator of greater social isolation which—alongside poverty—we conjecture increases the risk of becoming unclaimed upon death. Foreign-born status may be an important risk factor of social isolation and hence unclaimed mortality as well, particularly for undocumented migrants.^4,59^ On this dimension, Queens stands out with 47% foreign-born population, followed by Brooklyn (35.3%), the Bronx (34.2%), Manhattan (27.9%), and Staten Island (24.2%)^3,60^.

Hypothesizing that (lack of) economic and social resources play a key role in unclaimed mortality, the observed borough-level differences in poverty and percentages uninsured and foreign-born are noteworthy and clearly point to the Bronx, Brooklyn, and Queens as parts of the City with populations less equipped to weather a major public health crisis^4^ and more likely to become an unclaimed death. This prediction is consistent with our finding that the Bronx was the borough with by far the highest excess unclaimed mortality rates. Brooklyn and Queens had lower excess unclaimed death rates compared to the Bronx and these were similar to Manhattan, potentially reflecting greater (age) demographic vulnerability in all three boroughs, and in the case of Manhattan, higher levels of income inequality with pockets of deep disadvantage in some of its neighborhoods, particularly in northern Manhattan.^3^.

These findings have several public health policy implications. First, as shown here and in previous work^18^, unclaimed burials are strongly correlated with poverty. Federal emergency funding for burial assistance for families of those whose died of COVID-19 did not begin until the spring of 2021, a little over year after the pandemic began. Establishing timely, automatic emergency burial assistance during declared disasters could mitigate this burden and reduce the administrative lag that disproportionately affects low-income and immigrant households. In addition, more limited national burial assistance based on financial need during non-emergency times could help reduce the burden of these costs among the most economically disadvantaged. Secondly, during the peak of the pandemic, New York City’s mortuary and burial systems were overwhelmed, with assertions that the City applied shorter waiting times^37^ from death to determination of being unclaimed in order to relieve mortuary capacity usual constraints. To avoid this in the future, mortuary capacity during times of crisis, particularly when there is a strong possibility of mass casualties, should be expanded to ensure families have enough time to locate and properly grieve deceased relatives. Finally, these results suggest excess mortality was greater among economically disadvantaged and socially isolated populations. Taking a primary prevention approach, reducing excess mortality among vulnerable populations in future pandemics requires addressing the structural conditions that made the COVID-19 pandemic so deadly for these groups. For example, strengthening paid sick leave, eviction moratoria, better protections and benefits for undocumented individuals, and income supports could reduce the economic pressures that forced many low-income workers to return to work prematurely.^4,61^.

Continued research on the complex pathways that lead to unclaimed burial is needed to better inform future public health practice, particularly the effort to improve health equity. The higher concentration of unclaimed mortality in the poorer boroughs in general, and during the first wave of the pandemic in particular, further illustrates the need to better understand the nexus between economic deprivation and chronic health conditions in predominantly minority and immigrant neighborhoods. The fine-scale temporal and moderate-scale (borough) spatial data, on a particularly vulnerable population, used here illustrates how novel epidemiologic research design (that allow the isolation of “hidden” populations) can contribute to understanding mortality disparities during public health emergencies.

## Limitations

Our findings are subject to several limitations. First, some decedents (who would have been buried on Hart Island) may have instead been interred elsewhere – notably by charities or other organizations, particularly those that are religiously affiliated, thus reducing the estimate of socially isolated and/or economically disadvantaged decedents. This study focuses solely on unclaimed burials at Hart Island because a list of charity burials is not available and to our knowledge, the number of unclaimed burials conducted by charities is very small. Another notable exception impacts Staten Island (the city’s least populous borough), where the Office of the Public Administrator is responsible for public disposition of unclaimed deaths, a practice that has been in place for decades. (Since 2014, the borough’s Public Administrators’ office has partnered with a non-profit organization, the Foundation for Dignity^62^, which provides monetary assistance for burials for residents of the borough in private local cemeteries.) Since 2016, the borough maintains its own internet-based list of public burials^63^, and notably this list does not overlap with those interred on Hart Island. Information about public burials on Staten Island includes date of disposition (a process that can be long in some circumstances) not date of death which is the information that we use in this analysis, and thus deaths interred publicly on Staten Island are omitted from this analysis. Given that Richmond County’s long-standing practice of local public burial was operational prior to the pandemic and its procedures remained the same both before and after, we argue that the prior years of data on Staten Island residents in the Hart Island records act as a suitable counterfactual for excess mortality in 2020 for Staten Island residents buried on Hart Island. It may nevertheless place a little downward or upward pressure on the city-wide excess mortality estimates, depending on public burial rates on Staten Island as well, but given the much smaller population size of Staten Island, we expect the magnitude of its influence on the excess mortality of the city overall to be small.

Another limitation is that our assumptions about counterfactual deaths are imperfect, though we understand the general direction in which this limitation would influence our estimation. We estimate COVID-related excess mortality in 2020 using counterfactual unclaimed deaths based on 2019 levels either assuming constancy (our unadjusted results) or linear growth (our results adjusting for trends based on 2018 over 2019 changes), or the prior 5-year period in the regression-based estimation. Between Approaches A1 and A2, the trend-corrected estimates (A2) is preferred; and A3 is preferred to A2, though A2 is the most parsimonious approach and thus more transparent and readily replicable. All three yield very similar results, something we would not expect if any of these prior years were an outlier. Regardless, with these approaches we are concerned that the underlying assumption may be violated if, for example, any one year (or period within the year) had unusual levels (either higher or lower than expected) in unclaimed deaths. An important source of year-over-year and within-year mortality variation are influenza-related deaths. The 2017–2018 influenza season was unusually severe while the 2018–2019 season was more moderate: The city reported 2,004 deaths from influenza and pneumonia in calendar-year 2018 but only 1,624 in 2019.^64,65^ Furthermore, influenza and pneumonia mortality may have been higher in some boroughs (notably the Bronx) than others.^64^ The former implies that our estimate of the trend in unclaimed deaths (in A2) may be an underestimate (and more so in some boroughs). Because A3 uses a 5-year period and corrects for seasonal variation, it should be more robust and less affected by this plausible limitation.

There are two limitations that relate to how policy or behavior may have changed as a result of the pandemic, especially in the early stages. Some have noted that the medical examiner’s office applied shortened waiting times for disposition of bodies due to a much greater burden during the first months of COVID.^37^ This is a concern as it suggests that our excess mortality figures could be biased upwards, overstating the impact of COVID that we estimated. To address this, we sampled the Hart Island records for updates on several occasions since 2022 (both online and in person, looking at the original records which were still handwritten at the time). During those inquiries, we have found no evidence of an unusually high number of adjustments to the 2020 records such as decedents no longer being listed (online) or entries of reinterment (in the original recordkeeping books).

Another concern is that New York City may have experienced selective migration patterns in 2020 that might impact our excess mortality estimates. While the population of NYC grew in all boroughs from 2018, 2019, and 2020, Manhattan may have seen more out-migration of well-to-do New Yorkers and the Bronx may have seen lower rates of in-migration (from either domestic or international sources).^60,66^ These trends would mask to some small extent the true differences between the boroughs (making wealthier and poorer boroughs look more alike). In Manhattan, this would make the comparisons of death *rates* (but not ratios) in 2020 potentially somewhat greater, since the denominator grows potentially less than in other boroughs. (This would less likely impact the Bronx because reduced flows of migrant potentially impact both the numerator and denominator of any comparison of rates.)

There is a final limitation in that there is uncertainty in our knowledge about who becomes an unclaimed death – a limitation that is shared when studying this population more generally. Our analysis speaks to the impact of COVID on unclaimed mortality. As we argue, by the nature of becoming an unclaimed death, we capture individuals that, on average, are particularly socio-economically disadvantaged in life^15,18^, thus justifying using these data to examine vulnerable populations. Nevertheless, our analysis cannot identify exactly which vulnerability any given individual faced, or what statistical universe this population represents. Persons receiving public burial are not identified as a subpopulation in standard mortality records – like age, sex and race – and hence typically cannot be studied/compared at all with standard vital registration data alone since the necessary level of detail from the death certificate indicating burial location is not made available. With small changes in the way vital registration information is disseminated, this information could be made available, facilitating more systematic public health inquiry.

## Conclusion

This study is the first to show the impact of the initial wave of the COVID-19 pandemic on especially vulnerable New Yorkers, those whose receive a public burial. Public burial data are limited in some important ways but are underutilized in public health and social sciences to sharpen our understanding of who is disproportionately burdened by emerging disease. Because such data are publicly available and updated regularly^31^, they lend themselves to application in near real-time public health surveillance and targeted intervention, particularly of underserved and marginalized populations.

## Supplementary Information

Below is the link to the electronic supplementary material.

Supplementary Material 1

## Data Availability

The original Hart Island records can be accessed via a searchable online database maintained by the City of New York. The cleaned burial records analyzed in this paper are available as a STATA dta file in the figshare repository, https//doi.org/10.6084/m9.figshare.30087259. This file was also made available to the editors and reviewers as part of the submission. In addition, daily death data for the city of New York (including both those who were claimed and unclaimed) were provided by the New York City Department of Health and Mental Hygiene (DOHMH) Bureau of Vital Statistics. These data can only be accessed by authorized users, determined via an agreement between CUNY and DOHMH.
