# Supplementary material for: Deep disadvantage in mortality on the frontlines of the COVID-19 pandemic
Source: Sci Rep. 2026 Apr 7;16:16593. doi: 10.1038/s41598-026-41219-6 (PMC13219519; doi:10.1038/s41598-026-41219-6)
Supplement: Supplementary file 1 — Supplementary Material 1 [file 41598_2026_41219_MOESM1_ESM.docx]

**SUPPLEMENTARY MATERIALS**

**APPENDIX – METHODS**

Approach 3 (A3): Predicting counterfactual deaths in 2020 from 2015-2019 daily mortality data. In what we refer to as Approach 3 (A3) throughout the paper, we use a model-based approach to predict counterfactual mortality for the periods of interest (epi-year 2020, epi-weeks 10-15 in 2020, and epi-week 15 in 2020) based on daily death counts in epi-years 2015-2019. Specifically, we predict daily death counts in 2020 from count data regressions fitted to daily deaths (dependent variable) in epi-years 2015-2019. We report results from Poisson or more general negative binomial models (if there was overdispersion, which we found in the all-deaths data).

We make predictions using a large set of independent variables that capture year trends, within-year seasonality, daily weather conditions, federal holiday effects, and, when using death counts stratified by borough, borough effects and borough-specific trends. This strategy closely follows the approach used to model daily unclaimed deaths in the recent article titled “Extreme Weather and Mortality of Vulnerable Urban Populations: An Examination of Temperature and Unclaimed Deaths in New York City,” which is forthcoming in the journal *Demography*. The link to the paper (since it is not yet in press at the time of this writing) is available in the manuscript citation.

To obtain the counterfactuals for excess *unclaimed* deaths calculations that support the results in Tables 2 and 3 of the manuscript, we estimate models from Hart Island data using (1) all unclaimed deaths, (2) male deaths, (3) female deaths, and (4) deaths stratified by borough. The corresponding regression results are shown below as Appendix Table A1 (total, men, and women) and Table A2 (stratified by borough). To obtain the counterfactuals for excess deaths calculations for the city overall, supporting the results in Table 4, we estimate models from daily NYC deaths data using (1) all NYC deaths, (2) male deaths, and (3) female deaths. (We did not conduct this analysis by borough since we do not have access to daily NYC deaths by borough.) The corresponding all-deaths results are shown below as Appendix Table A3 (total, men, and women).

For each model we report McFadden R^2^ (for goodness-of-fit), the joint Wald test statistic and its p-value (for joint significance of coefficients on all predictors), and the log-likelihood (the logged probability of observing our data given the estimates). While the pseudo R^2^ values generally are in the single digits, indicating that our multivariate models provide only a modest improvement over a null model, the values are within the range commonly observed for complex count data models, where much of the variation may be due to unmeasured or random factors. The statistical significance of specific predictors (as indicated by joint Wald tests) suggests that our included variables nonetheless contribute meaningfully to explaining daily unclaimed death counts.

Since there was no evidence of overdispersion in the unclaimed deaths data, we use Poisson estimates for those outcomes (Tables A1 and A2). However, given overdispersion in total NYC deaths, we estimated negative binomial models for those outcomes and report the overdispersion parameter, alpha (variability relative to a Poisson process) and its standard error (Table A3).

**APPENDIX – TABLES**

**Count data regression model results tables (for Approach 3)**

**Table A1.** Results from count data regression models of daily unclaimed deaths in epi-years 2015-2019, Hart Island Deaths Combined, Men, and Women: Regression coefficients (robust standard errors)

| **Predictor** | **Total**  **Coefficient** | **(SE)** | **Men**  **Coefficient** | **(SE)** | **Women**  **Coefficient** | **(SE)** |
| --- | --- | --- | --- | --- | --- | --- |
| Year | 0.0469 | (0.0116) | 0.0461 | (0.0130) | 0.0552 | (0.0215) |
| ***Holidays*** |  |  |  |  |  |  |
| New Years Day | -0.1433 | (0.1902) | -0.6300 | (0.5289) | 0.4355 | (0.2658) |
| July 4^th^ | 0.0724 | (0.2773) | 0.1196 | (0.2650) | -0.4210 | (0.8782) |
| Veterans Day | -0.1138 | (0.2607) | 0.0074 | (0.3775) | -0.5211 | (0.9408) |
| Christmas | 0.0700 | (0.2254) | 0.0964 | (0.1733) | 0.0112 | (0.6158) |
| Thanksgiving | -0.3214 | (0.2612) | -0.2544 | (0.2804) | -0.5177 | (0.5536) |
| Memorial Day | 0.0046 | (0.2797) | 0.1182 | (0.3678) | -0.3189 | (0.5954) |
| Presidents Day | -0.1314 | (0.1491) | -0.1642 | (0.2764) | -0.4233 | (0.5544) |
| Labor Day | -0.4540 | (0.2548) | -1.0078 | (0.3744) | 0.3699 | (0.4139) |
| Columbus Day | -0.4831 | (0.3407) | -0.3416 | (0.2885) | -13.4806 | (0.4550) |
| ***Climate Variables*** |  |  |  |  |  |  |
| Maximum temperature | -0.0074 | (0.0050) | -0.0054 | (0.0054) | -0.0133 | (0.0083) |
| Minimum temperature | 0.0005 | (0.0043) | -0.0014 | (0.0047) | 0.0057 | (0.0075) |
| Total precipitation | -0.0691 | (0.0554) | -0.0745 | (0.0596) | -0.0509 | (0.0915) |
| ***Season (Ref. = Winter)*** | | | | | | |
| Summer | -0.3157 | (0.2976) | -0.1294 | (0.3331) | -0.8196 | (0.5378) |
| Spring | -1.0033 | (0.7200) | -1.0037 | (0.8111) | -0.8730 | (1.2832) |
| Fall | -0.3490 | (0.3206) | -0.2887 | (0.3611) | -0.5239 | (0.5760) |
| ***Temp. x Season Interactions*** |  |  |  |  |  |  |
| Max. temp. x Summer | 0.0050 | (0.0066) | 0.0003 | (0.0073) | 0.0165 | (0.0117) |
| Max. temp. x Spring | 0.0077 | (0.0112) | 0.0100 | (0.0121) | -0.0038 | (0.0193) |
| Max. temp. x Fall | 0.0092 | (0.0077) | 0.0057 | (0.0086) | 0.0193 | (0.0132) |
| Min. temp. x Summer | -0.0020 | (0.0063) | 0.0011 | (0.0073) | -0.0083 | (0.0114) |
| Min. temp. x Spring | 0.0068 | (0.0094) | 0.0032 | (0.0105) | 0.0217 | (0.0163) |
| Min. temp. x Fall | -0.0056 | (0.0072) | -0.0016 | (0.0081) | -0.0170 | (0.0122) |
| Constant Term | -93.2176 | (23.3155) | -92.0170 | (26.2637) | -111.2269 | (43.3517) |
| Log Likelihood | -3,273.9  48.05, p=0.001  0.01 | | -2,915.83 | | -1,831.81 | |
| Wald Test Statistic, P-value |  |  | 49.27, p=0.001 | | 929.71, p<0.001 | |
| McFadden R^2^ |  |  | 0.01 | | 0.01 | |

Number of observations (city-days) = 1,827. Coefficients are from Poisson regressions with robust standard errors in parentheses. The dependent variable is the number of individuals of the indicated sex group who died on a given day and were subsequently buried on Hart Island. This fitted model is used to predict the counterfactual death counts used in Approach 3 (A3) in Tables 2 and 3 (“NYC”).

**Table A2.** Results from count data regression models of daily unclaimed deaths in epi-years 2015-2019, Borough-level Hart Island deaths: Regression coefficients (robust standard errors)

| **Predictor** | **Coefficient** | **(SE)** |
| --- | --- | --- |
| Year | 0.0150 | (0.0242) |
| ***Borough (Ref. = Brooklyn)*** |  |  |
| Manhattan | -68.731 | (65.054) |
| Queens | -162.947 | (73.453) |
| Staten Island | 193.619 | (173.813) |
| Bronx | -62.191 | (62.231) |
| ***Year x Borough Interactions*** |  |  |
| Year x Manhattan | 0.0342 | (0.0323) |
| Year x Queens | 0.0807 | (0.0364) |
| Year x Staten Island | -0.0973 | (0.0862) |
| Year x Bronx | 0.0310 | (0.0309) |
| ***Holidays*** |  |  |
| New Years Day | -0.1432 | (0.2825) |
| July 4^th^ | 0.0724 | (0.2320) |
| Veterans Day | -0.1137 | (0.2503) |
| Christmas | 0.0700 | (0.3693) |
| Thanksgiving | -0.3214 | (0.3098) |
| Memorial Day | 0.0045 | (0.3267) |
| Presidents Day | -0.1314 | (0.2768) |
| Labor Day | -0.4540 | (0.4029) |
| Columbus Day | -0.4832 | (0.3857) |
| ***Climate Variables*** |  |  |
| Maximum temperature | -0.0074 | (0.0042) |
| Minimum temperature | 0.0005 | (0.0038) |
| Total precipitation | -0.0691 | (0.0520) |
| ***Season (Ref. = Winter)*** | | |
| Summer | -0.3154 | (0.2717) |
| Spring | -1.0031 | (0.6886) |
| Fall | -0.3479 | (0.3030) |
| ***Temp. x Season Interactions*** |  |  |
| Max. temp. x Summer | 0.0050 | (0.0060) |
| Max. temp. x Spring | 0.0077 | (0.0103) |
| Max. temp. x Fall | 0.0092 | (0.0072) |
| Min. temp. x Summer | -0.0020 | (0.0060) |
| Min. temp. x Spring | 0.0068 | (0.0087) |
| Min. temp. x Fall | -0.0056 | (0.0068) |
| Constant Term | -30.5778 | (48.8552) |
| Log Likelihood | -7,385.92 | |
| Wald Test Statistic, P-value | 784.66, p<0.001 | |
| McFadden R^2^ | 0.10 | |

Number of observations (borough-days) = 9,135. Coefficients are from Poisson regressions with robust standard errors in parentheses. The dependent variable is the number of individuals who died on a given day in a given borough and were subsequently buried on Hart Island. This fitted model is used to predict the counterfactual death counts used in Approach 3 (A3) in Table 3.

**Table A3.** Results from count data regression models of daily deaths in epi-years 2015-2019, NYC Deaths in Total, Men, and Women: Regression coefficients (robust standard errors)

| **Predictor** | **Total**  **Coefficient** | **(SE)** | **Men**  **Coefficient** | **(SE)** | **Women**  **Coefficient** | **(SE)** |
| --- | --- | --- | --- | --- | --- | --- |
| Year | 0.0035 | (0.0015) | 0.0093 | (0.0020) | -0.0024 | (0.0021) |
| ***Holidays*** |  |  |  |  |  |  |
| New Years Day | 0.0391 | (0.0388) | 0.0211 | (0.0500) | 0.0562 | (0.0438) |
| July 4^th^ | 0.0213 | (0.0425) | 0.0037 | (0.0669) | 0.0391 | (0.0363) |
| Veterans Day | -0.0366 | (0.0223) | -0.1074 | (0.0483) | 0.0286 | (0.0427) |
| Christmas | -0.0199 | (0.0256) | -0.0299 | (0.0376) | -0.0102 | (0.0427) |
| Thanksgiving | -0.0767 | (0.0243) | -0.1089 | (0.0355) | -0.0464 | (0.0283) |
| Memorial Day | -0.0035 | (0.0222) | -0.0440 | (0.0457) | 0.0359 | (0.0237) |
| Presidents Day | -0.0421 | (0.0424) | -0.0654 | (0.0401) | -0.0201 | (0.0493) |
| Labor Day | -0.0063 | (0.0239) | 0.0008 | (0.0413) | -0.0134 | (0.0378) |
| Columbus Day | 0.0547 | (0.0267) | 0.0940 | (0.0256) | 0.0142 | (0.0322) |
| ***Climate Variables*** |  |  |  |  |  |  |
| Maximum temperature | -0.0021 | (0.0007) | -0.0023 | (0.0009) | -0.0020 | (0.0008) |
| Minimum temperature | -0.0027 | (0.0006) | -0.0029 | (0.0008) | -0.0026 | (0.0008) |
| Total precipitation | 0.0121 | (0.0076) | 0.0142 | (0.0092) | 0.0101 | (0.0097) |
| ***Season (Ref. = Winter)*** | | | | | | |
| Summer | -0.1683 | (0.0407) | -0.1712 | (0.0554) | -0.1656 | (0.0487) |
| Spring | -0.4582 | (0.0965) | -0.4572 | (0.1248) | -0.4602 | (0.1285) |
| Fall | -0.1799 | (0.0485) | -0.2350 | (0.0656) | -0.1256 | (0.0620) |
| ***Temp. x Season Interactions*** |  |  |  |  |  |  |
| Max. temp. x Summer | 0.0026 | (0.0009) | 0.0024 | (0.0012) | 0.0028 | (0.0011) |
| Max. temp. x Spring | 0.0038 | (0.0014) | 0.0029 | (0.0019) | 0.0047 | (0.0018) |
| Max. temp. x Fall | 0.0025 | (0.0011) | 0.0035 | (0.0015) | 0.0014 | (0.0015) |
| Min. temp. x Summer | -0.0010 | (0.0009) | 0.0000 | (0.0012) | -0.0019 | (0.0012) |
| Min. temp. x Spring | 0.0023 | (0.0011) | 0.0040 | (0.0015) | 0.0006 | (0.0015) |
| Min. temp. x Fall | 0.0000 | (0.0010) | -0.0002 | (0.0014) | 0.0001 | (0.0014) |
| Constant Term | -1.6973 | (3.0721) | -14.1814 | (4.1082) | 9.3827 | (4.1794) |
| Overdispersion Parameter (α) | 0.0016 | (0.0003) | 0.0015 | (0.0005) | 0.0018 | (0.0005) |
| Log Likelihood | -7,355.45 | | -6,618.78  456.20, p<0.001  0.030 | | -6,637.91 | |
| Wald Test Statistic, P-value | 931.08, p<0.001 | |  |  | 653.13, p<0.001 | |
| McFadden R^2^ | 0.050 | |  |  | 0.041 | |

Number of observations (city-days) = 1,827. Coefficients are from negative binomial regressions with robust standard errors in parentheses. The dependent variable is the number of individuals of the indicated sex group who died on a given day. These fitted models are used to predict the counterfactual death counts used in Approach 3 (A3) in Table 4.

**Extended Results Tables: Tables 2-4 shown in the main text with columns for Standard Errors and 95% Confidence Intervals**

This is a version of Table 2 shown in the main text that includes Standard Errors (“SE”) and Confidence Intervals (“95% CI”). Rows showing results related to 2020 are highlighted in grey for emphasis. D_t,t-1_=Difference in death counts or rates (t minus t-1). Approach 1 (A1): D_20-19_=2020-2019. Approach 2 (A2): D^Adj^_20-19_=Linear-trend adjusted difference in deaths counts or rates (D_20-19_-D_19-18_). Approach 3 (A3): D^Reg^=2020 Observed - 2020 Regression-Predicted. Rates are per 100,000 Residents. A1: R_20/19_=Ratio of 2020 over 2019 observed death counts. A2: R^Adj^_20/19_=Linear-trend adjusted ratio (2020 observed over 2019 observed plus D_19-18_). A3: R^Reg^=Regression-adjusted ratio (2020 observed over 2020 regression-predicted). The fitted models underlying A3 are shown in Appendix Table A1. Since epi-year 2020 (a leap year) has 53 epi-weeks while 2019 and 2018 only have 52 epi-weeks, the denominators in epi-year 2020 rate calculations are adjusted by a factor 1+1/53. Epi-week 10 to 35 in 2020 is March 1 to August 29. Epi-week 15 in 2020 is Sunday April 5 to Saturday April 11, the week unclaimed deaths peaked.

This is a version of Table 3 shown in the main text that includes Standard Errors (“SE”) and Confidence Intervals (“95% CI”). Rows showing results related to 2020 are highlighted in grey for emphasis. D_t,t-1_=Difference in death counts or rates (t minus t-1). Approach 1 (A1): D_20-19_=2020-2019. Approach 2 (A2): D^Adj^_20-19_=Linear-trend adjusted difference in deaths counts or rates (D_20-19_-D_19-18_). Approach 3 (A3): D^Reg^=2020 Observed - 2020 Regression-Predicted. The fitted models underlying A3 are shown in Appendix Table A1 (“Total” model for “NYC Overall”) and Table A2 (borough-level model for “Borough”). Rates are per 100,000 Residents. Since epi-year 2020 (a leap year) has 53 epi-weeks while 2019 and 2018 only have 52 epi-weeks, the denominators in epi-year 2020 rate calculations are adjusted by a factor 1+1/53. Epi-week 10 to 35 in 2020 is March 1 to August 29. Epi-week 15 in 2020 is Sunday April 5 to Saturday April 11, the week unclaimed deaths peaked.

This is a version of Table 4 shown in the main text that includes Standard Errors (“SE”) and Confidence Intervals (“95% CI”). Rows showing results related to 2020 are highlighted in grey for emphasis. D_t,t-1_=Difference in death counts or rates (t minus t-1). Approach 1 (A1): D_20-19_=2020-2019. Approach 2 (A2): D^Adj^_20-19_=Linear-trend adjusted difference in deaths counts or rates (D_20-19_-D_19-18_). Approach 3 (A3): D^Reg^=2020 Observed - 2020 Regression-Predicted. Rates are per 100,000 Residents. A1: R_20/19_=Ratio of 2020 over 2019 observed death counts. A2: R^Adj^_20/19_=Linear-trend adjusted ratio (2020 observed over 2019 observed plus D_19-18_). A3: R^Reg^=Regression-adjusted ratio (2020 observed over 2020 regression-predicted). The fitted models underlying A3 are shown in Appendix Table A3. Since epi-year 2020 (a leap year) has 53 epi-weeks while 2019 and 2018 only have 52 epi-weeks, the denominators in epi-year 2020 rate calculations are adjusted by a factor 1+1/53. Epi-week 10 to 35 in 2020 is March 1 to August 29. Epi-week 15 in 2020 is Sunday April 5 to Saturday April 11, the week unclaimed deaths peaked.
